# Prevalence and determinants of stunting in a conflict-ridden border region in Armenia - a cross-sectional study

**DOI:** 10.1186/s40795-017-0204-9

**Published:** 2017-12-02

**Authors:** Arin A. Balalian, Hambardzum Simonyan, Kim Hekimian, Richard J. Deckelbaum, Aelita Sargsyan

**Affiliations:** 1 Fund for Armenian Relief of America, #22 Khorenatsi Street, Yerevan, Armenia; 2 Department of Epidemiology, Columbia University, Mailman School of Public Health, Room #1616, 722 W 168th Street, New York, NY 10032 USA; 3 Institute of Human Nutrition, Columbia University, 630 W. 168th Street, New York, NY 10032 USA

**Keywords:** Stunting, Conflict, Armenia, Infant and child nutrition, Growth

## Abstract

**Background:**

Despite global efforts, stunting remains a public health problem in several developing countries. The prevalence of stunting among 0- to 5-year-old children in Armenia has increased from 17% in 2000 to 19% in 2010. A baseline study was conducted among preschool children in Berd, a region near the northeastern border of Armenia that has experienced intermittent military tension for over 20 years.

**Methods:**

We conducted a cross-sectional study including 594 children aged 6-month- 6 years old and their caregivers in our analysis, to assess the prevalence and determinants of stunting. We calculated the anthropometric measurements and hemoglobin levels of children; analyzed children’s stool and conducted a survey with children’s caregivers. We employed the hierarchical logistic regression model to explore the predictors of stunting among 25–72 months old children and multivariable logistic regression models to investigate the predictors of stunting among 6–24 months old children. Individual and residence level variables were included in the models including anemia, minimum dietary diversity, mothers’ height, the overall duration of breastfeeding, birthweight, child’s history of diarrhea and mean socio-economic score.

**Results:**

The prevalence of stunting was significantly higher among the 6–24 months old children (13.3%) compared to the children aged 25–72 months old (7.8%). We did not find any differences in the prevalence of stunting by place of residence in either age group. The 6–24 months old children who consumed at least four food groups during the previous day (minimum dietary diversity) had 72% lower odds of being stunted (*p* < 0.05). Each kilogram increase in birthweight was associated with 76% lower odds of being stunted (OR = 0.24, *p* < 0.01). Mother’s height significantly decreased the odds of stunting among the children 25–72- months old (OR = 0.86, *p* < 0.001). BMI was also a significant predictor of stunting among both age-groups.

**Conclusions:**

The study results highlight the significance of mother’s height, birthweight, and adequate complementary feeding to reduce stunting. Further studies are needed to determine the possible association of anemia and stunting with the ongoing conflict in the region, as well as socioeconomic conditions and food insecurity in the region.

## Background

Several studies have found that protracted conflicts have an adverse impact on health [1–3]. Current on-going conflicts in the former Soviet states, such as Ukraine, and in the nearby Middle East certainly have an impact on childhood health [4, 5]. Armenia is one such conflict-ridden former Soviet country, located in the Caucasus region. Long-standing military tensions may exacerbate health and nutrition status of vulnerable populations affected by such circumstances, particularly young children [6].

According to the World Health Organization (WHO), 54% of all child deaths are associated with undernutrition [7]. Despite global efforts to achieve the Millennium Development Goals, the percentage of stunted children under age five remains high. Since 1990, there has been a steady decrease in stunting worldwide; however, it is estimated that globally in 2016, 24% of children under age five were still stunted [8, 9]. Stunting during childhood can lead to reduced immunity, increased susceptibility to non-communicable diseases, impaired physical and mental development, and reduced productivity [10].

Several factors influence stunting, including the socioeconomic status of the household, parents’ education, anemia, soil-transmitted helminths (STH) infections, diarrheal infections, dietary intake and complementary feeding [11–22]. Short overall duration of breastfeeding, as well as the late introduction of appropriate solid foods, have both been associated with stunting in many studies [18, 23–26] Birth outcomes such as low birth weight and birth length are also important factors that are associated with higher prevalence of stunting [24, 27, 28].

In a study conducted in Sri Lanka, maternal height was inversely related to the prevalence of stunting [18]. Low birthweight was associated with higher rates of stunting in the same study. Socioeconomic status of the family was associated with stunting in among school children in Nigeria [29]. In another study conducted in Nigeria, children who did not receive timely complementary feeding and children with less than minimum food diversity had higher odds of being stunted. [22].

There are limited data regarding infant and child nutrition status in the Republic of Armenia. The Armenian Demographic and Health Survey (ADHS), conducted every 5 years, is the primary source of information regarding nutrition and child growth patterns. According to the 2010 ADHS, the prevalence of stunting among 0- to 5-year-old children increased to 19% from 17% in 2000 [16]. A recent study conducted in Yerevan, the capital of Armenia, identified the prevalence of stunting as 17.9% among 5- to 17-month-old children, which is close to the 2010 ADHS estimated percentage of around 17.3% in Yerevan [16, 30].

According to the ADHS, the prevalence of stunting in 2010 was 16.1% in the Tavush province [16] which was one of the regions with the highest prevalence of stunting in Armenia. Frequent tensions and skirmishes along the Armenian–Azerbaijani border have increased in recent years [31–33], resulting in diminished agricultural capacity and affecting socioeconomic conditions in this region. Tensions are intense in the Berd region in particular, because of its proximity to the border between Armenia and Azerbaijan.

The growing trend of stunting in Armenia necessitates a thorough investigation to understand its risk factors to inform policies and interventions to reverse the trend. Although the determinants of stunting have been well investigated in several studies, the specific precursors of stunting might vary among different countries and regions [34]. This different pattern is evident among former Soviet countries [30, 35]. Therefore, it is important to study the risk factors responsible for nutritional problems of children specific to Berd region in Armenia.

The main aim of this study was to determine the prevalence and determinants of stunting among preschool children in Berd city and its seven surrounding rural communities, a vulnerable border region in the Tavush province in the Republic of Armenia, which is itself a low-middle income country. We specifically explored if there are significant differences in the prevalence of stunting among the children 6–24 months and 25–72 months old. We also examined if the prevalence of stunting in each age group was different by place of residence. We further investigated the influence of anemia, soil-transmitted helminths, feeding practices, dietary intake, maternal and household characteristics and birth outcomes on stunting among children 6–24 months and 25–72 months old.

## Methods

### Study design

This cross-sectional study included anthropometric (height, weight) measurements, blood tests to measure hemoglobin (HgB) levels of 6-month to 6-year-old children, and stool analysis for intestinal STH in 12-months to 6-year-old children. The survey also included a questionnaire for caregivers (mainly mothers) to understand the determinants of malnutrition in the target communities. The study was designed and conducted from September 2013 to March 2014.

### Study population

The study population for the anemia analysis and anthropometric measurements included all 6-month to 6 year-old children living in the city of Berd and seven surrounding rural communities (Chinchin, Varagavan, Paravakar, Verin Caghkavan, Nerkin Karmiraghbyur, Tavush, and Aygepar). The only exclusion criteria were if the child was diagnosed with a blood coagulation or neurological disease; however, no child in our sample met the exclusion criteria.

### Population size and sampling method

A representative sample of children from Berd city aged 6 months to 6 years old was randomly selected to participate in the study (*n* = 352). Study participants were chosen from a sample frame consisting of all children living in Berd city, as registered in the Tavush province’s administrative records. The children from Berd were selected based on a stratified random sampling strategy. We chose age groups (year) as the strata. The number of the samples within each age group (stratum) were calculated separately based on $\mathrm{n}=\frac{{\mathrm{Z}}^2\mathrm{P}\left(1\hbox{-} \mathrm{P}\right)}{{\mathrm{d}}^2}$ equation to estimate population prevalence [36, 37]. Since the total number of children in the rural communities was small, the study team included all children and caregivers from these areas (*n* = 491).

### Data collection

Healthcare providers in rural and urban primary healthcare facilities (family physicians and laboratory physicians and nurses) were trained in the study protocols and assigned as study coordinators in their regions. The adherence to study protocols by coordinators and staff was monitored through frequent spot-checks. The anthropometric measurements, blood and stool analysis, and surveys with the parents/caregivers took place in the primary healthcare facilities in Berd and the local rural communities. The study coordinators obtained a written informed consent from the caregivers of all participants. After collecting the child’s anthropometric measurements, the caregiver participated in a survey administered by the physician or nurse. The survey questionnaire included questions about maternal and child characteristics, infant feeding practices, birth outcomes and child’s dietary intake, measured by 24-h recall.

### Measurements

The HgB levels of the children were measured using HemoCue® HB 301, a U.S. Food and Drug Administration (FDA)-approved device designed for quick analysis of HgB in capillary, venous, or arterial blood [38–41]. As recommended by the WHO for STH surveys, we conducted stool tests on samples from 12-months to 6-year-old children from the Berd region [42].

The Kato-Katz method was used to perform the stool analyses [42]. This technique is utilized to diagnose STH, such as *Ascaris lumbricoides,* and *Trichuris trichiura,* in stool samples [43]. We provided stool container kits to parents to collect the stool samples. Parents were instructed to collect the first morning stool of their children. They were also instructed to refrigerate the stool samples and transport them to the nearest local healthcare facility within 12 h of collection.

We adapted the WHO, and UNICEF Infant and Young Child Feeding (IYCF) practices questionnaire to assess feeding practices as well as maternal and child characteristics in the target region [42, 44]. These questions were translated and validated for use in the ADHS 2010 [16]. We made minor changes to the questionnaire to gain information on maternal education, in addition to the history of soil-transmitted helminths disease, anemia, and diarrhea. The primary health care physicians administered the after the anthropometric measurements, and blood samples were collected by the nurses.

Children’s weight was measured using electronic scales. The children’s height (length in recumbent status for children younger than 24 months) was measured with appropriate measuring boards or stadiometers by the trained primary health care providers in the study sites.

### Study variables

The primary outcome variable was stunting, analyzed as a dichotomous outcome. Stunting was considered positive for all the children whose height-for-age was below -2SD compared to the median height-for-age of the WHO growth standards [45].

Anemia, one of the explanatory variables, was considered positive for the children with blood HgB levels lower than 110 g/L. Altitude adjustments were made to the HgB values of the children living in areas more than 1000 m above sea level [46]. The presence of STH confirmed via stool laboratory analysis was another explanatory variable.

The survey questionnaire administered to caregivers asked about child’s birth weight and birth length; child’s age; any breastfeeding; exclusive breastfeeding; breastfeeding duration; residence; mother’s height; mother’s participation in community training about child nutrition; accessibility of printed materials about child nutrition; any history of prolonged diarrhea or STH reported by the caregiver; and a detailed dietary intake assessment of the child during the last 24 h.

We used a standardized socio-economic score as a composite of (a)mothers’ education; (b) the financial status of the family compared to neighboring households; (c)food insecurity. Further, we calculated the mean socioeconomic status score based on the place of residence. The information about birth weight, birth length and birthdate were confirmed based on administrative records.

To assess the effect of conflict on stunting, we created a variable based on the geographical distance from the border and front lines of the conflict zone. The two villages closest to the front line were considered positive for being exposed to violence.

Minimum dietary diversity, another explanatory variable, was created based on the scoring system of the WHO and UNICEF indicators [44] for the assessment of infant and young child feeding practices. It included seven food groups (grains, legumes, dairy, flesh, eggs, Vitamin A-rich foods, and other fruits and vegetables). Children who had consumed at least four of the seven groups were scored as positive. Children whose birthweight were less than 2500 g were considered having a low birthweight as suggested by WHO and UNICEF [43].

### Data management and statistical analysis

The data were double entered using SPSS 21.0 and analyzed using SAS 9.4 statistical software. We calculated frequencies and proportions to describe categorical variables and computed means and standard deviations for continuous variables. We used Chi-Square and ANOVA to assess the intergroup comparison for children 6–24 months and 25–72 months old separately. We further examined whether the association between stunting and main predictor variables was modified by place of residence using Breslow-Day test.

To estimate the determinants of stunting among children 6–24 months old and children 25–72 months old separately, we first fitted a univariable logistic regression model with stunting as the outcome variable (Table 1). Next, we selected all the variables associated with stunting at the level of *p* < 0.25 as well as the clinically significant variables to fit the multivariable logistic regression and hierarchical models (Table 1) [47]. The associations with *p* < 0.1 and *p* < 0.05 were considered marginally statistically and statistically significant.

**Table 1:** Univariable Logistic Regression Analysis Results for the association between explanatory variables and stunting

Variable Name	6–24-months old children		25–72-months old children	
	OR	95% CI	OR	95% CI
Birth weight (kilograms)	0.47	[0.20–1.08]	0.61	[0.31–1.22]
Low birthweight (<2500 g) [Yes/No(Ref)]	2.06	[0.52–8.19]	1.88	[0.62–5.74]
Birth length (centimeters)	0.88	[0.76–1.02]	0.96	[0.80–1.15]
Body mass Index	1.31^*^	[1.06, 1.62]	1.16^*^	[1.01, 1.34]
Breastfeeding duration (months)	1.06	[0.98–1.15]	1.05^*^	[1.00–1.10]
Child’s history of diarrhea [Yes/No(Ref)]^a^	3.21^*^	[1.15–8.95]	1.42	[0.52–3.86]
Caregiver’s participation in community training programs [Yes/No(Ref)]	0.56	[0.12–2.60]	0.39	[0.12–1.32]
Child’s gender [male/female(ref)]	0.86	[0.35–2.14]	2.42^*^	[1.1–5.31]
Child’s age (months)	1.10^*^	[1.01–1.20]	0.99	[0.96–1.01]
Mother’s height (cm)	0.91^*^	[0.84–0.99]	0.89^**^	[0.84–0.96]
Anemia [Yes/No(Ref)] ^b^	0.48	[0.18–1.29]	1.93	[0.75, 4.95]
Minimum dietary diversity [Yes or No(Ref)] ^c^	0.45	[0.18–1.11]	0.78	[0.37–1.65]
Exclusive breastfeeding reported by mother [Yes/No(Ref)] ^d^	1.13	[0.45–2.80]	0.89	[0.38–2.13]
Composite standardized SES score ^e^	0.82	[0.52–1.29]	1.53	[0.85, 2.74]
Child’s history of iron medication intake [Yes/No(Ref)]	2.27	[0.43–12.01]	1.12	[0.25–5.01]
Child’s previous history of soil-transmitted helminth disease [Yes/No(Ref)]	<0.001	[0–999.99]	0.94	[0.40–2.23]
Soil-transmitted helminth (lab analysis)	1.51	[0.46–5.0]	1.34	[0.61–2.91]
Negative (ref)	1.00		1.00	
Ascaris lumbricoides	2.27	[0.56, 9.23]	0.93	[0.27,3.26]
Trichuris trichiura (whipworm)	0.85	[0.10, 7.24]	1.80	[0.73, 4.42]
hookworms	–		<0.001	[0–999.99]
pinworms	<0.001	[0–999.99]	<0.001	[0–999.99]
Residence [urban/rural(Ref)]	1.14	[0.46–2.85]	1.51	[0.74–3.11]
Financial condition of the family compared to neighbors ^f^				
Significantly below average (Ref)	1.00		1.00	
Below average	0.83	[0.22, 3.14]	1.34	[0.31, 5.85]
Average	0.42	[0.13, 2.41]	1.94	[0.56, 6.72]
Above average	0.26	[0.03,2.46]	2.62	[0.49, 13.91]
Significantly above average	0.45	[0.04, 4.5]	4.2	[0.62, 28.35]
Maternal Education				
> highschool (10 years) (Ref)	1.00		1.00	
≤ highschool (10 years)	1.22	[0.49, 3.01]	0.87	[0.42, 1.83]

*CI* Confidence Interval, *OR* Odds Ratio, *Ref* Reference category

^a^Caregivers answered to the following question:” Has your child ever had any episodes of prolonged recurrent diarrhea?”

^b^Positive if the child had age and altitude adjusted anemia

^c^Positive if children had consumed at least four of the seven food groups

^d^ Positive if the child had been exclusively breastfed during the first 6 months of his/her life

^e^ Socio-economic score as a composite of (a)mothers’ education; (b)Family income compared to their neighboring households; (c)whether the child has ever slept hungry because of food unavailability based on mothers’ answer to those questions

^f^Caregivers were asked about the Financial condition of the family compared to neighboring households

To build our final models, we first explored the association between the dependent and independent variables using binary logistic regression for both age groups using the LOGISTIC procedure in SAS. We further fitted the data for each age group separately conducting multilevel logistic regression analysis by proc. glimmix procedure in SAS, assuming the place of residence as the group variable and the highest level of the hierarchy.

The estimated G matrix was not positive definite for the unconditional model estimating predictors of stunting among children 6–24 months old. Therefore, we used the multivariable logistic regression model to estimate the predictors of stunting among children in this age group.

To estimate the predictors of stunting for the children 25–72 months old, we used the multilevel logistic regression approach. Utilizing the Likelihood ratio test (LRT) and Akaike’s Information Criterion (AIC) we compared the hierarchical models to estimate the predictors of stunting among 25–72 months old children and to examine the improvement in model fit.

Model A was the multivariable logistic regression model including all the individual level predictors adjusted by age and gender of the child. Model B was the multilevel logistic regression model assuming variance components (VC) G matrix for all the residence groups and including only the random intercept to estimate the effect of a typical residence place level effect. In model C, we included only the individual level variables to account for the crude effects of these variables in the presence of random intercept. Model D included anemia as another individual level predictor. Subsequently, we included the residence-level mean socio-economic composite score variable in model E to explain the residence level variation in stunting. However, we did not observe a significant association between stunting and the residence level mean socio-economic composite score, and the inclusion of the variable did not improve the model fit. Model F included all the variables in model D, adjusted for age and gender. Finally, model G included all the variables in model E, adjusted for age and gender. We refrained from including random slope since it did not significantly improve the model fit when comparing to model F using LRT test.

The multivariable logistic regression models to estimate the predictors of stunting among 6–24 months old children were tested and compared by the Hosmer–Lemeshow goodness-of-fit test, AIC and LRT and Nagelkerke’s R^2^. The multivariable logistic regression analysis resulted in six consecutive models. The final model included all the statistically and clinically significant variables that had the highest Hosmer–Lemeshow test score and pseudo R^2^ to predict the prevalence of stunting. Model A includes crude unadjusted predictors of stunting including birthweight, child’s history of any diarrhea reported by the caregiver, and minimum dietary diversity. Model B included all the individual level variables in model A adjusting for age of the child by months and gender of the child. We included anemia, standardized socio-economic score, and presence of soil-transmitted helminth infection in each of the three consecutive models subsequently. However, including the variables did not contribute to the model fit.

## Results

In total 843 children were selected to participate in the study. The overall response rate was 80% (*n* = 674). We did not include children who were older than 6 years or younger than 6 months in the analysis. The final analysis included 594 children and their caregivers after excluding missing observations. Of the total number of children included in the study, 46.52% were girls, and 53.48% were boys; 34.43% were from Berd city, and 65.57% were from the rural communities named in methods above. Most of the caregivers included in the study (61.2%) had at least a high school level of education (≥10 years of education). More than 66.22% considered the financial status of their household to be average or above average compared to their neighbors. The prevalence of low birth weight, stunting, and anemia was significantly higher among the children aged 6–24 months compared to the children 25–72 months old. The prevalence of minimum dietary diversity was significantly higher among 25–72 months old compared to the 6–24 months old children. There were no significant differences in the prevalence of STH among younger and older children (Table 2). The most common STH found in the stool analysis were *Ascaris lumbricoides* and *Trichuris trichiura.*

**Table 2:** Demographic and baseline characteristics of study participants overall and by age group

			Child’s age		
	Categories	Total (*N* = 604)	6–24 months old	25–72 months old	*p* value^a^
Demographic characteristics					
Gender, n (%)^*^					
Male		323 (53.48)	81 (48.79)	242 (55.25)	0.15
Female		281 (46.52)	85 (51.20)	196 (44.75)	
Location, n (%)					
Berd		208 (34.43)	63 (37.95)	145 (33.11)	0.26
Rural Areas		396 (65.57)	103 (62.05)	293 (66.89)	
Baseline characteristics					
Birthweight, mean (SD), Kilograms		3.15 (0.50)	3.14 (0.52)	3.16 (0.50)	0.67
Birthlength, mean (SD), centimeters		49.74 (2.24)	49.57 (2.64)	49.80(2.07)	0.27
Breastfeeding duration, mean (SD), months		11.46 (7.16)	9.92 (5.67)	12.04 (7.58)	0.00
Mother’s height, mean(SD), centimeters		159.46 (5.84)	159.83 (5.78)	159.33(5.87)	0.35
Composite SES score, mean (SD)		8.88 (1.60)	8.99(1.73)	8.83(1.55)	0.30
Body Mass Index, mean (SD)		16.72 (2.22)	17.73 (2.17)	16.34 (2.12)	0.00
Maternal Education, n (%)					
≤ highschool (10 years)		370 (61.26)	98 (59.04)	272 (62.1)	0.49
> highschool (10 years)		234 (38.74)	68 (40.96)	166 (37.9)	
Financial condition of the family compared to neighbors					
Significantly below average		89 (14.74)	23 (13.86)	66 (15.07)	0.22
Below average		115(19.04)	32 (19.28)	83 (18.95)	
Average		336 (55.63)	86 (51.81)	250 (57.08)	
Above average		42 (6.95)	15 (9.04)	27 (6.16)	
Significantly above average		22 (3.64)	10 (6.02)	12 (2.74)	
Child’s history of diarrhea, n (%)					
Yes		73 (12.2)	25(15.15)	48 (11.09)	0.17
No		525 (87.8)	140 (84.85)	385 (88.91)	
Caregiver’s participation in community training programs, n (%)					
Yes		107 (18.20)	23 (14.11)	84 (19.27)	0.22
No		490 (81.80)	140 (85.89)	350 (80.28)	
Exclusive breastfeeding reported by mother, n (%)					
Yes		199 (39.10)	90 (56.25)	220 (63.04)	0.14
No		310 (60.90)	70 (43.75)	129 (36.96)	
Child’s history of sleeping hungry reported by caregiver, n (%)					
Always		3 (0.50)	0	3 (0.69)	0.55 ^*b*^
Often		10 (1.68)	4 (2.44)	6 (1.38)	
Sometimes		69 (11.56)	21 (12.80)	48 (11.09)	
Never		515 (86.26)	139 (84.76)	376 (86.84)	
Child’s history of iron medication intake, n (%)					
Positive		31 (5.16)	8 (4.85)	23 (5.52)	0.80
Negative		568 (94.84)	157 (95.15)	411(94.48)	
Child’s previous history of soil-transmitted helminth disease, n (%)					
Positive		97 (16.19)	4 (2.42)	93 (21.43)	0.00
Negative		502 (83.81)	161 (97.58)	341 (78.57)	
Minimum Dietary Diversity, n (%)					
Positive		414 (68.54)	97 (58.43)	317 (72.37)	0.00
Negative		190 (31.46)	69 (41.57)	121 (27.63)	
Soil-transmitted helminth confirmed by Kato Katz method, n (%)					
Negative		469 (78.69)	142 (86.59)	327 (75.58)	0.49 ^*c*^
Ascaris lumbricoides		57 (9.56)	12 (7.32)	45 (10.37)	
Trichuris trichiura (whipworm)		67 (11.25)	9 (5.49)	58 (13.59)	
hookworms		1 (0.17)	0	1(0.23)	
pinworms		2 (0.33)	1(0.61)	1 (0.23)	
Stunting, n (%)					
Positive		56 (9.32)	22 (13.33)	34 (7.80)	0.03
Negative		545 (90.68)	143 (86.67)	402 (92.20)	
Anemia, n (%)					
Positive		115 (19.07)	69 (41.57)	46 (10.53)	0.00
Negative		488 (80.93)	97 (58.43)	391 (89.47)	

Abbreviations; *SD* standard deviation

^a^ *p* values for the differences between child age groups for continuous variables were obtained using an analysis of variance *F*-test. *P* values for different proportions between child age groups for categorical/binary variables were obtained by Chi-squared test

^b^ *p* values for different proportions between child age groups and whether the child has ever slept hungry reported by the caregiver was calculated by Fisher’s exact test

^c^ *p* values for different proportions between child age groups and parasite types among the children who tested positive for soil-transmitted helminths

We further explored the association between stunting, anemia and minimum dietary diversity and each age group to see if it was modified by place of residence. The prevalence of stunting, anemia, minimum dietary diversity and STH, stratified by age-group and place of residence are summarized in Table 3. The association of stunting with the age-groups did not differ significantly across the place of residence. We did not find any evidence of effect modification of the association by place of residence (Berd or rural areas) (Table 3).

**Table 3:** Demographic and baseline characteristics of study participants overall and by age group stratified by place of residence

			Child’s age						
	Categories	Total (N = 604)	Urban (*N* = 208)			Rural (*N* = 396)			*p* value^a^
			6–24- month old	25–72 month old	OR	6–24- month old	25–72 month old	OR	
Stunting, n (%)									
Positive		56 (9.32)	9 (14.28)	14 (9.67)	1.56	13 (12.75)	20 (6.87)	1.98	0.68
Negative		545 (90.68)	54 (85.72)	131 (90.33)		89 (87.25)	271 (93.13)		
Anemia, n (%)									
Positive		115 (19.07)	20 (31.75)	13 (8.97)	4.72^***^	49 (47.57)	33 (11.30)	7.12^***^	0.73
Negative		488 (80.93)	43 (68.25)	132 (91.03)		54 (52.43)	259 (88.70)		
Minimum Dietary Diversity, n (%)									
Positive		414 (68.54)	43 (68.25)	112 (77.24)	1.58	54 (52.43)	205 (69.97)	2.11^**^	0.50
Negative		190 (31.46)	20 (31.75)	33 (22.76)		49 (47.57)	88(30.03)		
Soil-transmitted helminth confirmed by Kato Katz method, n (%)									
Positive		130 (21.70)	3 (1.44)	26 (17.93)	0.22^**^	19 (18.81)	82 (28.28)	0.59	0.16
Negative		469 (78.30)	60 (95.24)	119 (82.07)		82 (81.19)	208 (71.72)		
Low Birthweight n (%) (Birthweight < 2500 g)									
Yes		45 (7.58)	3 (4.84)	7 (4.83)	1.00	10 (9.80)	25 (8.77)	1.13	0.88
No		549 (92.42)	59 (95.16)	138 (95.17)		92 (90.20)	260 (91.23)		

Abbreviations; *OR* Odds Ratio

^a^ *p* values for Breslow-Day test for Homogeneity of the Odds Ratios of different proportions between child age groups and selected variables stratified by place of residence (Urban vs. Rural)

^*^ *p* < 0.05

^**^ *P* < 0.001

^***^ *P* < 0.0001

As previously described in data management and statistical analysis, we fitted a multivariable logistic regression model among children between 6 and 24 months to identify the determinants of stunting among this age group (Table 4). Model A shows a significant effect of any diarrhea reported by the caregivers on stunting (OR = 3.85, *p* < 0.05). However, when this variable was adjusted for age and gender of the child in model B, the effect estimate significance was reduced (OR = 2.83 *p* < 0.10). In model B, the children who had consumed at least four food groups (minimum dietary diversity) during the previous day of the investigation had significantly lower odds of stunting (OR = 0.28, *p* < 0.05).

**Table 4:** Estimated predictors of Stunting among 6–24- month old Children

	Model A	Model B	Model C	Model D	Model E
	OR (95%CI)	OR (95%CI)	OR (95%CI)	OR (95%CI)	OR (95%CI)
Effects Estimates					
Gender					
Male		0.82 (0.28, 2.42)	0.81 (0.27, 2.4)	0.80 (0.39, 2.95)	0.82 (0.28, 2.41)
Female (Ref)		1.00	1.00	1.00	1.00
Age		1.25 ^**^ (1.11, 1.41)	1.25^***^(1.10, 1.41)	1.25^***^ (1.10, 1.41)	1.24^***^ (1.10, 1.40)
BMI	1.30^*^ (1.05, 1.63)	1.55^***^ (1.2, 2.01)	1.55^***^(1.20, 2.01)		
Birthweight, Kilograms	0.40 (0.16, 1.03)	0.24^**^ (0.08, 0.71)	0.26^*^ (0.08, 0.80)	0.20^**^ (0.06, 0.62)	0.24^**^ (0.08, 0.70)
Child’s history of diarrhea					
Yes	3.85^*^ (1.27, 11.69)	2.83 (0.83, 9.66)	2.88 (0.84, 9.86)	2.56 (0.73, 8.92)	2.93 (0.85, 10.07)
No (Ref)	1.00	1.00	1.00	1.00	1.00
Minimum Dietary Diversity					
Positive	0.58 (0.22, 1.55)	0.28^*^ (0.09, 0.87)	0.29^*^ (0.09, 0.91)	0.27^*^ (0.09, 0.88)	0.28 ^*^ (0.09, 0.87)
Negative (Ref)	1.00	1.00	1.00	1.00	1.00
Standardized socioeconomic status score			0.87 (0.50, 1.51)		
Anemia					
Positive				0.34 (0.10, 1.12)	
Negative(Ref)				1.00	
Soil-transmitted helminth confirmed by Kato Katz method, n (%)					
Positive					1.36 (0.34, 5.45)
Negative (Ref)					1.00
Model goodness of fit					
-2logL	111.801	95.708	95.391	92.274	95.524
AIC	121.801	109.708	111.391	108.274	111.524
Hosmer Lemeshow test	0.84	0.79	0.81	0.43	0.53

Abbreviations: *Ref* Reference Category, *OR* Odds Ratio, *CI* Confidence Interval

Model A: Multivariable logistic regression Model adjusted for age and gender; Model B: Model A adjusted for age and gender; Model C: Model B + standardized socio-economic score; Model D: Model B + anemia; Model E: Model B + Soil-transmitted helminth in stool confirmed by Kato Katz

^*^ *P* < 0.05 ^**^ *P* < 0.01 ^***^ *P* < 0.001

Also in model B, each kilogram increase in birth weight was associated with 76% lower odds of being stunted (OR = 0.24, *p* < 0.01). BMI also appeared to be a significant predictor of stunting in model B. The odds of stunting increased by 55% with each unit increase in BMI score (OR = 1.55 *p* < 001). Finally, with each increasing month of age, the children had higher odds of being stunted (OR = 1.25, *p* < 0.001). Therefore, significant predictors of stunting in children age 6–24 months were minimum dietary diversity, birthweight, BMI, and age.

We employed a hierarchical modeling approach to estimate the predictors of stunting among the 25–72 months old children (Table 5). The effect estimates remained similar across the six models. Model B includes the random intercept only and illustrates the odds of stunting for a typical child living in a typical community. The ICC value calculated for this model indicates that approximately 12% of the variability in stunting prevalence among children is accounted for by residence level characteristics and the rest is explained by individual level characteristics. Model C includes the crude estimates of the predictors of stunting that were used in multivariable logistic regression model A (Table 5). The odds of stunting seemed to increase by 6% with each month increase in the overall duration of breastfeeding in model C. Nevertheless; this association appeared to fade away when the model was adjusted for age and gender of the child. In model F, the odds of stunting were significantly greater for boys compared to the girls (OR = 2.5 *P* < 0.05) and maternal height appeared to be protective against stunting. The odds of stunting seemed to decrease by 13% with each centimeter increase in maternal height (OR = 0.86, *p* < 0.001). Finally, with each unit increase in the child’s BMI the odds of stunting were increased by 26% (OR = 1.26, *p* < 0.01) (Table 5). In model G, we added the residence level mean socioeconomic score, however, adding this variable did not improve the model fit.

**Table 5:** Estimated predictors of Stunting among 25–72- month old Children

	Model A	Model B	Model C	Model D	Model E	Model F	Model G
	OR (95%CI)	OR (95%CI)	OR (95%CI)	OR (95%CI)	OR (95%CI)	OR (95%CI)	OR (95%CI)
Fixed Effects Estimates							
Gender							
Male	2.36^*^ (1.03,5.38)					2.5^*^ (1.05, 5.9)	2.47^*^ (1.04,5.86)
Female (Ref)						1.00	1.00
BMI	1.22^*^ (1.04, 1.42)		1.25^**^(1.07, 1.47)	1.25^**^(1.07, 1.47)	1.26^**^ (1.07, 1.48)	1.26^**^ (1.06, 1.49)	1.26^**^ (1.06, 1.5)
Age	0.98 (0.96, 1.01)					0.99 (0.96, 1.01)	0.99 (0.96, 1.01)
Breastfeeding duration (months)	1.05^*^ (1.00, 1.10)		1.06^*^ (1.00, 1.10)	1.05^*^ (1.00, 1.11)	1.05^*^ (1.00, 1.11)	1.04 (0.99, 1.10)	1.04 (0.99, 1.1)
Mother’s height (centimeters)	0.87^***^ (0.81, 0.94)		0.87 ^***^ (0.81, 0.94)	0.86 ^***^ (0.80, 0.94)	0.87^***^ (0.81,0.94)	0.87^***^(0.81,0.94)	0.86^***^(0.79,0.93)
Anemia							
Positive				2.28(0.79, 6.61)	2.39 (0.82, 6.94)	2.19 (0.75, 6.39)	2.30 (0.79, 6.72)
Negative(Ref)				1.00	1.00	1.00	1.00
Residence level variables							
Mean Socioeconomic score					2.06 (0.70, 6.1)		2.13 (0.67, 6.7)
Error Variance							
Residence Level Intercept (SE)		0.47 (0.51)	0.54 (0.57)	0.50 (0.53)	0.45 (0.50)	0.54 (0.56)	0.39 (0.48)
Model goodness of fit							
-2logL	202.571	235.75	204.69	202.59	199.97	197.34	194.79
AIC	214.571	239.75	214.69	214.59	213.97	213.34	212.79
ICC		0.12	0.14	0.13	0.12	0.11	0.13

Abbreviations: *SE* Standard Error, *ICC* Intraclass Correlation Coefficient, *Ref* Reference Category, *OR* Odds Ratio, *CI* Confidence Interval

Model A: Multivariable logistic regression Model adjusted for age and gender

Model B: Unconditional model with random intercept

Model C: Multilevel logistic regression model

Model D: Model C + anemia

Model E: Model D + residence level mean socioeconomic score variable

Model F: Model D + adjusted for gender and age

Model G: Model E + adjusted for gender and age

^*^ *P* < 0.05 ^**^ *P* < 0.01 ^***^ *P* < 0.001

## Discussion

Our study found that the prevalence of stunting was higher among children 6–24 months old in both Berd and the rural areas compared to children aged 25–72 months old in the same region. The higher proportion of stunting and anemia among children 6- to 24-months old might be related to recent increases in border conflict in this area (Table 3). The lower prevalence of stunting in children 25–72 months old could be explained by the catch-up growth phenomenon [48, 49].

We found that minimum dietary diversity was significantly and negatively associated with stunting among children 6–24 months old. These findings strengthen the argument that factors such as inadequate complementary feeding and low minimum dietary diversity might be the most critical areas to address in public health interventions to decrease the prevalence of stunting, particularly among children aged 6 to 24 months. Moreover, the preventive effect of minimum dietary diversity on stunting (see Tables 4 and 5) is similar to other studies conducted in the Republic of Armenia and other countries [30, 50, 51].

We also found that the higher the birth weight (analyzed as a continuous variable), the lower the odds of stunting among 6–24 months -old children, which is consistent with the literature [11, 52–54]. Nevertheless, there was not a statistically significant association between low birth weight (dichotomous) and stunting in the final multivariable model, as is often seen in the literature [18, 24, 28]. This could be because the prevalence of low birth weight in this age group was low (9.8% [95%CI 3.9, 15.6] in rural areas and 4.8% [95%CI 0.6, 10.3] in Berd). Our findings suggest that analyzing birthweight as a continuous variable might be more appropriate.

The association between BMI and stunting among children was one of the novel findings of our study. We found that the odds of stunting increased significantly among children in both age-groups with each unit increase in BMI adjusting for other predictors in our models (Tables 4 and 5). This finding could indicate that stunting and overweight can be experienced in the same individuals. In fact, the double burden of malnutrition is a rising problem in low and middle-income countries. [55]. Although we could not establish the sequential events in the development of stunting and BMI trajectories in our study population, it is hypothesized that stunted children are more predisposed to become overweight. This association has already been explored in several studies [56–59]. This phenomenon is also explained by dietary consumption patterns which are poor in animal protein and high in carbohydrates and fats [57]. We found that minimum dietary diversity was lower among 6–24- month old children. This finding could explain the significant association of BMI with stunting in our study population.

We found that, with each centimeter increase in the mother’s height, the odds of stunting among all children aged 25–72 months old decreased by 13%. This finding, which is consistent with the literature, indicates the possible role of genetics and the intergenerational effect of malnutrition on the development of stunting [18, 60].

We also found that the odds of stunting among boys were 2.36 times the odds of stunting among girls in the 25–72 months old. Several studies conducted predominantly in Sub-Saharan Africa have found similar results [61–63]. This finding could be explained by the higher vulnerability of male children towards early life adversities in comparison with females [63–65]. Particularly, boys were more likely to need advanced life support than girls in a study conducted among preterm and low-birthweight children [64]. In another study conducted among the low-birthweight infants, girls were more likely to survive to 5 years of age than the males [65].

The overall prevalence of anemia was high in our study population. However, anemia was not significantly associated with stunting in univariable and multivariable models among any of the age groups. The prevalence of anemia was significantly higher among the 6–24 months old children compared to 25–72 months old children in both the rural and urban areas. A possible explanation for this finding could be the lower dietary diversity among 6–24 months old children compared to children aged 25–72 months (Table 3).

A history of long episodes of diarrhea was only associated with stunting among the 6–24 months old children in the multivariable logistic regression. However, the association faded away after adjusting for age and gender and in hierarchical models. In fact, diarrhea was found to be associated with stunting in similar studies [66–69]. During episodes of diarrhea, children lose water and micronutrients, which can result in malnutrition if not adequately replaced. We recognize that this variable was subjective and “long episodes of diarrhea” was not well defined for the survey respondents. This finding, nonetheless, suggests that history of diarrhea may be a significant predictor of stunting among 6–24 months old children, and better measurements of the history of diarrhea in future studies could further explore this relationship.

The positive effect of overall duration of breastfeeding (months) on the development of stunting among 25–72 months old children, although very small (OR = 1.05), may indicate inadequate complementary feeding, which is also supported by the finding of low minimum dietary diversity. It could also indicate the inadequate frequency of breastfeeding during the day; however, the frequency of breastfeeding was not measured in this study.

This is the first study in this restive border region assessing the trends and determinants of stunting. The study included the entire community in the rural areas (census), which indicates that the statistical inferences were accurate for the rural regions and the representative random sample from the Berd region also increases the generalizability of the results. The authors pretested the questionnaire along with study coordinators in the region to minimize the possibility of measurement errors.

Nevertheless, there were several limitations in this study. The questionnaire utilized for the survey with mothers lacked questions regarding the water and sanitation situation of the respondents’ households. Although our instrument assessed the diet of the children based on 24-h recall questions, we do not exclude the possibility of recall bias. Recall bias may also have affected responses to other subjective questions like those related to diarrhea and breastfeeding duration. The stool analysis was also only conducted once and not in a modern laboratory. However, this misclassification would be non-differential. We also did not include a separate question to assess the frequency and level of border conflict in each study site in our survey. We did not find a significant association between residence-level mean socioeconomic score and stunting in our hierarchical models. We acknowledge that our questionnaire did not capture accurate information to calculate the socio-economic score for individuals. Only three variables were measuring the socioeconomic status of the study participants’ households. Furthermore, our questionnaire did not assess the family income and father’s occupation accurately, which could help us to determine the family’s socio-economic position better.

## Conclusions

This study identified several risk factors that are associated with the development of stunting among children in the conflict-ridden border regions of Armenia. Factors such as minimum dietary diversity and history of previous episodes of diarrhea indicate the necessity of adequately feeding children as well as the management of the diarrheal disease. The findings of this study will contribute to the development of appropriate interventions targeting timely complementary feeding in vulnerable regions to reduce stunting. Although anemia was not associated with stunting, the prevalence of anemia was very high among the 6–24 months old children and should be a focus of dietary intake interventions to promote optimal growth patterns among children in the region. Further research should consider a more thorough questionnaire to capture the socioeconomic status of the study participants, to determine the association between anemia and stunting, and to further assess the effect of the ongoing conflict in the region. The findings of this study can inform public health programmers and policymakers to focus on promoting appropriate complementary feeding, dietary diversity, and management of diarrhea for optimal growth among young children in this region.

## Abbreviations

ADHS: Armenian Demographic and Health Survey
AIC: Akaike’s Information Criterion
CI: Confidence Interval
FDA: United States Food and Drug Administration
HgB: Hemoglobin
ICC: Intraclass Correlation Coefficient
LRT: Likelihood ratio test
SD: Standard Deviation
STH: Soil-transmitted Helminths
VC: Variance Components
WHO: World Health Organization
